# Irrational use of antimalarial drugs in rural areas of eastern Pakistan: a random field study

**DOI:** 10.1186/1471-2458-12-941

**Published:** 2012-11-01

**Authors:** Shafaat Yar Khan, Ahmad Khan, Muhammad Arshad, Hafiz Muhammad Tahir, Muhammad Khalid Mukhtar, Khawaja Raees Ahmad, Najma Arshad

**Affiliations:** 1 Department of Biological Sciences, University of Sargodha, Sargodha, Pakistan; 2 Department of Zoology, University of Punjab, Lahore, Pakistan

**Keywords:** Malaria, *Plasmodium*, Eastern Pakistan, Antimalarial drugs, Mosquito vectors, Diagnosis, Blood Examination Rate (BER)

## Abstract

**Background:**

Prescription of antimalarial drugs in the absence of malarial disease is a common practice in countries where malaria is endemic. However, unwarranted use of such drugs can cause side effects in some people and is a financial drain on local economies. In this study, we surveyed the prevalence of malaria parasites in humans, and the prevalence of the malaria transmitting mosquito vectors in the study area. We also investigated the use of antimalarial drugs in the local people. We focused on randomly selected rural areas of eastern Pakistan where no malaria cases had been reported since May 2004.

**Methods:**

Mass blood surveys, active case detection, passive case detection, and vector density surveys were carried out in selected areas of Sargodha district from September 2008 to August 2009. Data pertaining to the quantities and types of antimalarial drugs used in these areas were collected from health centers, pharmacies, and the district CDC program of the Health Department of the Government of the Punjab.

**Results:**

Seven hundred and forty four blood samples were examined, resulting in a Blood Examination Rate (BER) of 3.18; microscopic analysis of blood smears showed that none of the samples were positive for malaria parasites. Investigation of the mosquito vector density in 43 living rooms (bedrooms or rooms used for sleeping), 23 stores, and 32 animal sheds, revealed no vectors capable of transmitting malaria in these locations. In contrast, the density of *Culex* mosquitoes was high. Substantial consumption of a variety of antimalarial tablets, syrups, capsules and injections costing around 1000 US$, was documented for the region.

**Conclusion:**

Use of antimalarial drugs in the absence of malarial infection or the vectors that transmit the disease was common in the study area. Continuous use of such drugs, not only in Pakistan, but in other parts of the world, may lead to drug-induced side effects amongst users. Better training of health care professionals is needed to ensure accurate diagnoses of malaria and appropriate prescription of antimalarial drugs delivered to communities.

## Background

Malaria is a major vector borne disease that is the second most prevalent disease in Pakistan. Its transmission is seasonal, with epidemic outbreaks in Baluchistan, Khyber Pakhtoon Khawa (KPK) and Sindh province. It is predominantly a problem in the Federally Administrated Tribal Areas (FATA) and along the border of Iran and Afghanistan. However, in Punjab, the most populous province, it is less common and the disease incidence is much lower than in other areas of Pakistan. The disease is transmitted by the bite of an infected female mosquito belonging to the genus Anopheles. A total of 577 species of Anopheles mosquitoes have been recorded in the world, of which 77 species act as vectors for malaria. In Pakistan, about 24 Anopheles species are known; of these only two are malaria vectors (*Anopheles culicifacies* and *An. stephensi*)*.* Two new species, namely, *An. fluviatilis* and *An. annularis* are suspected of transmitting malaria in Baluchistan province [1].

Common symptoms of malaria include high fever, shivering, anemia, joint pain, vomiting and retinal damage. Health care professionals generally use these symptoms to diagnose malaria [2]. In addition, all pyrexia of unknown origin (PUO) with or without the above mentioned symptoms are generally treated as malaria when patients do not respond to antibiotics (and in the absence of a laboratory confirmed diagnosis).

For effective malaria control in Pakistan, professional bodies must work within clear public health guidelines, have adequate funding, and the support of opinion leaders, especially the government [3]. Two species of malaria parasite, *Plasmodium vivax* and *P. falciparum* cause malaria in Pakistan. *P. vivax* is the most common species, constituting approximately 76% of diagnosed cases, out of 104, 454 confirmed cases, while the remaining 24% are due to *P. falciparum*, which causes the most dangerous form of the disease [1,4].

A large number of anti-malarial drugs are used to treat malaria all over the world. However, dose-related side effects include vomiting, nausea, (quinine, Mefloquine) fatigue, anorexia, dizziness, pulmonary toxicity (Mefloquine), neuropsychiatric effects (Mefloquine and Chloroquine), effects on the retina, blindness, (quinine), and neurotoxicity targeting mainly the auditory and vestibular pathway (artemisinin in combination with Mefloquine) [5-12]. Inappropriate use of antimalarial drugs by clinicians, general practitioners and health facilities is a common practice in many developing countries like Pakistan [13-15]. The problem is mainly related to a lack of training, and an absence of proper treatment guidelines and diagnostic facilities, especially in rural areas. Therefore, in areas where clinicians have no access to laboratory facilities, treatment is based mainly on clinical symptoms, which leads to overdiagnosis of malaria and excessive use of antimalarial drugs [16]. Mannan et al., [17] reported that 22.4% of patients treated with antimalarial drugs at health facilities in Khartoum, Sudan, did not present with fever, nor reported an attack of fever before presenting themselves for treatment; in addition, 35.4% of patients treated with antimalarials had malaria parasite negative blood films. Irrational prescription of antimalarial drugs, without laboratory confirmation of malarial disease, has also happened in Lahore, the second largest district of Pakistan (population > eight million); this observation provided the impetus to conduct the present study. While working for the communicable disease control program in the Lahore district, no evidence of malarial vectors or parasites were recorded (unpublished data) in most parts of the district; nevertheless, antimalarial use was observed on a large scale.

The objective of the present work was to study the prevalence of malaria, the vectors that transmit it, and the use of antimalarial drugs in randomly selected rural areas of the Sargodha district in the eastern part of the country that has seen no malaria cases since May 2004 (monthly surveillance data of CDC, Punjab 2004–08).

## Methods

### Site selection

The selected area for the study was the union council (UC) # 125 in the Sargodha district of Pakistan. This UC is a thickly populated rural area comprising six villages with a total population of 23,359. This area is a vast agricultural landscape with an organized irrigation system consisting of channels, canals and tributaries. This UC is a central point for the surrounding areas and therefore has a good number of health facilities, consisting of one public sector Rural Health Centre (RHC), two private hospitals, 4 clinics with physicians and 20 quacks. A quack is an unqualified person that works in a rural area and has experience of working with a physician, after which he starts to prescribe or sell medicines to people. He has no diagnostic skills, nor access to diagnostic facilities. Quacks often work in areas where people have no qualified health care personal available to them or are too poor to pay a qualified physician. All the health facilities mentioned above do not have microscopes or rapid diagnostic tests for malaria diagnosis except RHC, where facility of microscopy was available but cannot be used because of a lack of trained staff.

This area was randomly selected for study from areas where no cases have been reported for more than four years according to the monthly surveillance data from the CDC Program, Health Department Government of the Punjab. The present study is a small-scale preliminary study conducted in a small geographical area that has not been investigated previously.

### Blood sampling

Three methods of blood sampling were used to collect blood samples for detection of human malaria parasites:

i. Mass Blood Survey (MBS).

ii. Passive Case Detection (PCD) at health facilities.

iii. Active Case Detection (ACD) through house-to-house visits.

**Figure 1:**
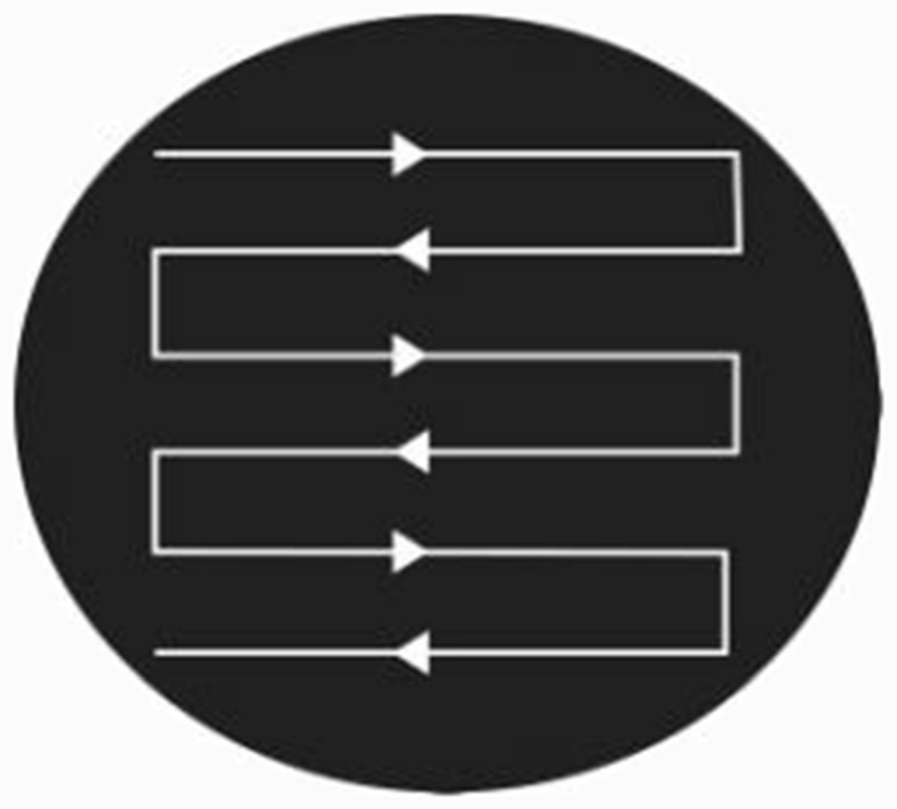
The pattern of movement of slide during blood examination.

Blood samples were collected from September 2008 to August 2009. For MBS, 9 Government and 12 private schools were selected from the six villages within the UC. The blood samples were collected from all of the children and students who had a fever or history of fever attacks with symptoms of chills, headache and other malaria symptoms during the previous month. ACD was carried out by house-to-house visits to collect blood samples from suspected malaria cases. All houses were visited in the area for this purpose. Health facilities were visited regularly (average twice a week) during the study period for PCD. We obtained the assistance of health care professionals, health facility and clinic owners, and quacks, which was needed for blood sample collection from patients who attended the facility to seek treatment for fevers with chills and headache (pyrexia of unknown origin). The sample size was calculated at a 95% confidence level with a 5% confidence interval at the worst disease incidence of 0 to 3.535. Through use of the above sampling methods, we were able to keep the study area under continuous surveillance during the entire study period. This ensured that blood smears were collected from all of the suspected cases of malaria, alongside those cases that were diagnosed and treated by the physicians, health care providers, and quacks. The blood films were prepared and examined using standard World Health Organization (WHO) protocols for detection of *Plasmodium* spp. Slides were screened using a 100× objective, and 100 fields examined for the presence of malaria parasites The method used to screen the slides (i.e., the pattern of movement used by each of the slide readers) is shown in Figure 1. The slides were read three times, starting with scientists with expertise in microscopy who read the slides twice, each times using a different reader that was blind to the results obtained by the first reader. In addition, senior microscopists from the Provincial Reference Laboratory of the CDC program in the Punjab performed an additional blind reading. The Blood Examination rate (BER) was calculated using the following formula as provided by TAMA and DOMC [1], where BER = total slides made/total population × 100.

### Vector density

To study the vector density in the selected area, adult mosquito collections were carried out in the morning, twice a month in all of the six villages. Mosquitoes were collected from bedrooms that had been slept in the previous night (insecticide treated bed nets were not used in these rooms or houses), animal sheds, and stores following standard WHO protocols and guidelines [18]. A pyrethrum spray catch method was used with white spreadsheets [19]. Although the method is suitable for collecting mosquitoes, it causes a lot of disruption to the household occupants during such collections. Mosquito identification was carried out using the identification key of National Institute of Malaria Research and Training (NIMRT) Lahore, a sub office of National Institute of Health, Pakistan.

### Data on antimalarial drug use

Data about the types and doses of anti-malarial drugs used for the treatment of malaria in the area were collected. To achieve this, a data analysis tool was designed. Twelve pharmacies were included in the study. The owners of these pharmacies were interviewed to obtain data about the quantity of antimalarial drugs used in that area as they were the main source of antimalarial drugs for the population. Data about the antimalarial drugs that were sold at each pharmacy or dispensed by practitioners during the study period were also collected. At the start of the study, the pharmacy owners were requested to provide information regarding the sales volumes of the different types of antimalarial drugs (i.e., tablets, capsules, syrup, and injectable drugs). Pharmacy owners were requested to provide regular updates on a monthly basis during the study period. The same data were collected from health facilities and the public sector, i.e., from the RHC and district CDC program, both of which were purchasing antimalarial drugs from the government medical store depot. The ethics committee department of the Biological Sciences University of Sargodha duly approved the study and its design.

## Results

### Malaria parasite incidence

A total of 744 blood samples were collected (496 males, 248 females); from this, the blood examination rate (BER) of the total study population was calculated 3.18. The microscopy results showed no malaria parasites in the population; therefore, the malaria Parasite Incidence (PI) during the 12 months study period was zero (Table 1). Among those that attended the health facility, nobody was tested for malaria using Rapid Diagnostic Test (RDT) methods or microscopy because no facilities were available for this. All such individuals were treated as malaria cases and were prescribed antimalarial drugs. The antimalarials mainly prescribed to them were Chloroquine, Sulfadoxine-pyrimethamine, Artemether and Lumefantrine- Artemether.

**Table 1:** Detail of blood samples and their result for malaria microscopy in rural areas of Sargodha Pakistan

**Sr. No.**		**Sampling site**	**No. Of samples**	**Gender**		**Results**		BER*
				**Male**	**Female**	***P.falciparum***	***P. vivax***	
**1**	**ACD/MBS**	Village 46 SB	261	164	97	0	0	3.08
**2**		Village 115 SB	75	58	17	0	0	1.57
**3**		Village 58-A(SB)	75	52	23	0	0	2.37
**4**		Village 58B (SB)	36	24	12	0	0	1.14
**5**		Village 59 SB	40	31	9	0	0	1.38
**6**		Village 47 SB	25	18	7	0	0	2.71
**7**	** P CD**	Health Facilities	232	149	83	0	0	-
**Total**			**744**	**496**	**248**	**0**	**0**	**3.18**
	** P CD**	**Age wise breakup of 232 PCD study subject ( who visited health facilities)**						
		Less than 1 year	5	4	1	0	0	-
		1-5 years	33	23	10	0	0	-
		6-13 years	79	51	28	0	0	-
		14 & above	115	71	44	0	0	-

*Sr.No*: Serial Number.

*BER*: Blood Examination Rate.

*ACD*: Active Case Detection.

*MBS*: Mass Blood Survey.

*PCD*: passive case detection.

### Vector density

Vector density studies were carried out in all of the six villages in the study area. Living rooms (43), stores (23), and animal sheds (32) were screened for the presence of *Anopheles* mosquitoes. Mosquito density was highest in the animal sheds, followed by the stores, then the living rooms. No malarial vectors were found in any of the above three types of dwellings. However, Culex mosquitoes were collected from such places. The ratio of male to female *Culex* spp. calculated is provided in Table 2.

**Table 2:** **Vector density data of *Anopheles *mosquitoes in rural areas of Sargodha Pakistan**

**Sr. No.**	**Site of collection (Village)**	**No. of rooms**			**No. of *Anopheles*/Room**												**No. *Culex *spp./Room**					
		LR*	S**	AS***	***A. culicifacies***						***A. stephensi***						**Male**			**Female**		
					**Male**			**Female**			**Male**			**Female**								
					**LR**	**S**	**AS**	**LR**	**S**	**AS**	**LR**	**S**	**AS**	**LR**	**S**	**AS**	**LR**	**S**	**AS**	**LR**	**S**	**AS**
1	46 SB	17	8	9	0	0	0	0	0	0	0	0	0	0	0	0	273	513	1321	197	222	464
2	115 SB	7	4	4	0	0	0	0	0	0	0	0	0	0	0	0	381	311	607	146	143	115
3	58-A SB	5	3	5	0	0	0	0	0	0	0	0	0	0	0	0	217	276	593	139	65	114
4	58-B SB	4	3	4	0	0	0	0	0	0	0	0	0	0	0	0	129	271	297	44	28	133
5	59 SB	5	2	6	0	0	0	0	0	0	0	0	0	0	0	0	393	85	511	87	36	237
6	47 SB	5	3	4	0	0	0	0	0	0	0	0	0	0	0	0	131	175	179	66	53	97
	**Total**	**43**	**23**	**32**	**0**	**0**	**0**	**0**	**0**	**0**	**0**	**0**	**0**	**0**	**0**	**0**	**1524**	**1631**	**3508**	**679**	**547**	**1160**

*Sr. No*, Serial Number.

* LR, Living room.

**S, Store.

***AS, Animal Shed.

### Data on antimalarial drug use

Table 3 shows the data for antimalarial drugs used. The types and formulations of the drugs sold and used in the study area included, tablets (Chloroquine, Sulfadoxine-pyrimethamine, Amodiaquine), syrups (Chloroquine, Sulfadoxine-pyrimethamine, Amodiaquine), capsules (Artemether, Lumefantrine-Artemether), and injections (Artemether and Chloroquine). The total cost calculated for the above antimalarials was PKR. 31,831/-(approximately 400 US$), 24,460/-(approximately 300 US$), 15,520/-(approximately 200 US$) and 8500/- (approximately 100 US$), tablets, syrups, capsules and injections respectively. The above figures were calculated based on the price of single tablet, syrup, capsule or injection multiplied by the total number of the item sold or consumed, and then summarized by the cost of each group separately.

**Table 3:** Detail of antimalarials used in rural areas of district Sargodha Pakistan

**Antimalarial**	**Tablet**	**Syrup**	**Capsule**	**Injection**
Chloroquine	16000	500	0	140
Sulfadoxine-pyrimethamine	3450	340	0	0
Amodiaquine	2150	315	0	0
Artemether	0	0	332	30
Lumefantrine Artemether	0	0	90	0
**Total**	**21600**	**1155**	**422**	**170**
**Cost ( PKR)**	**31,831/-**	**24, 460/-**	**15, 520/-**	**8500/-**
**Cost (Approximate US $)**	400*	**300**	**200**	**100**

* 1 US$ = 81.5 PKR (Forex conversion rate in Aug 2009).

## Discussion

Malaria in Pakistan has low to high endemicity. Areas with high endemicity include FATA, Baluchistan and KPK, whilst malaria endemicity in the Punjab is relatively low [1]. The major factors underlying the high malaria endemicity in FATA, Baluchistan and KPK include, for example, variations in seasonal malaria transmission, drought, hydrological change, population movement, drug resistant parasites and insecticide resistant vectors.

As malaria can be fatal, the general use of antimalarial drugs in malaria endemic countries is common. Before the Roll Back Malaria (RBM) program was implemented, the presumptive treatment of PUO cases remained a component of national policy implemented throughout country, including the Punjab. However, with the launch of the RBM program in 2003, the emphasis changed to one of its component “Early diagnosis and Prompt treatment” of malaria as part of the national health policy. According to National Treatment Guidelines, antimalarials should be given or prescribed after diagnosis, and presumptive treatment should be avoided as it may lead to parasite resistance and possible treatment side effects. However, whether effective communication of this policy to health providers and pharmacies has been successful is an open question. Hence, malaria is still overdiagnosed and excessive use of antimalarial drugs continues in the areas where there is no access to laboratory facilities to obtain an accurate diagnosis of malaria [16].

General and specific blood surveys along with vector density studies are the key tools for better surveillance of malaria. Use of such approaches, would direct malaria control strategies (i.e. proper use of antimalarial drugs and target-oriented use of insecticides) to the areas that need them. Such data would help health authorities to prioritize and allocate scare resources appropriately so that better health facilities are available in areas that need them. This is particularly important in third world countries where resources are scarce.

In this study, we chose an area of Pakistan, in the Sargodha district, to evaluate the use of antimalarials in relation to the prevalence of malaria. Use of insecticide treated bed nets is not practiced in the area, being an area with no malaria case history since 2004. Similarly, no insecticide spraying has been carried out in the area since that time. However, people do use various kinds of mosquito repellents to avoid being bitten by mosquitoes.

During the present study, *Plasmodium* spp were not observed in any of the blood films from the sampled population. However, microscopy does have limitations for detection of parasites in areas with very low parasite prevalence. The BER was 3.18 for this area, a value that is higher than the value recommended for surveillance purposes [1]. No cases of malaria were recorded in this area during the study period. These results are in accordance with data from the CDC program of the Health Department, Government of Punjab, wherein no cases of malaria have been reported since May 2004. However, our study was not intended to determine if malaria had been eliminated from the area, which could only be determined by a more extensive survey.

Consistent with the above findings, no *An. culicifacies* or *An. stephensi* mosquitoes were found in any of the areas sampled in the six villages. However, relatively large numbers of *Culex* mosquitoes were found. Among the *Culex* species collected, the ratio of male to female mosquitoes was very high. *Culex* densities were very high in animal sheds compared to stores and living rooms, which may reflect the feeding preference of these mosquitoes. In contrast, absence of Anopheline mosquitoes at the sampling sites might suggest that ecological factors, such as habitat competition (e.g., competitive exclusion) might exist. The use of pyrethrum spray for catching mosquitoes is a good method for collecting endophilic mosquitoes at rest indoors and provides good estimates of the mean mosquito density per house in a given area. However, it will miss mosquitoes that leave houses immediately after feeding, or include those that enter after feeding outdoor on another host. Anopheline mosquitoes in this area are not known to be resistant to pyrethroid insecticides; therefore, the absence of malaria vectors in the area during the study period provides important evidence to support the lack of parasites observed on blood films during the study period (PI =0).

Our data revealed that a considerable quantity and range of antimalarial were used to treat misdiagnosed malaria. Tablets constituted the largest number of antimalarials sold or used (21,600), whilst syrups (1155), capsules (422), and injections (170) were sold or used less frequently. The total amount spent by the local population on antimalarials was 79, 311/-PKR (approximately 1000$). This amount is almost equal to the total annual medical budget of a single basic health unit, which is a primary health care facility in this country.

In the absence of malaria in the study area, the use of such large quantities of antimalarial drugs is irrational and particularly unjustified when there are no vectors that can transmit the disease. Not only was there no need for such medicines, they are expensive and may cause side effects in some people. The present study reflects the need for training to improve malaria diagnosis among health care professionals, as well as increasing awareness in the population. In particular, prescribing antimalarials injections without a confirmed laboratory diagnosis must be discouraged. Injections are very effective when they are prescribed properly, but may cause harm when used inappropriately [20].

## Conclusion

We conclude that, even taking into account the limitations of this small study, the high use of antimalarial drugs reported by the participating health facilities and pharmacies contrasts sharply with the lack of malarial disease and vector in the area during the study period. This type of practice needs to be redressed as it is causing economic loses and may lead to toxicity issues. There is a strong need to assess the malaria situation in such areas with the help of laboratory diagnosis as well as the vector presence at a large scale, not only to this part of the world, but in other places as well. Hence there is a need for increased training of health care professionals. Whilst looking for new malaria treatments and control strategies, it is also important to ensure that resources are not used to treat misdiagnosed malaria. Public awareness campaigns may help to educate people about malaria; however, surveillance remains an important tool in areas where malaria could still pose a threat to public health.

## Competing interests

The authors declare that they have no competing interests. Moreover they do not have affiliation and financial interest with any company/organization whose product appears prominently in the manuscript. There was no source of funding for this study.

## Authors’ contributions

All the authors have equal contribution to this study, have read, and approved the final manuscript.

## Pre-publication history

The pre-publication history for this paper can be accessed here:

http://www.biomedcentral.com/1471-2458/12/941/prepub
